# Treatment of refractory lupus nephritis using leflunomide: A prospective study

**DOI:** 10.3389/fimmu.2023.1133183

**Published:** 2023-03-17

**Authors:** Shuo Zhang, Yiran Chen, Xin Chen, Yan Zhao, Xiaofeng Zeng, Fengchun Zhang, Li Wang, Mengtao Li

**Affiliations:** ^1^ Department of Rheumatology and Clinical Immunology, Peking Union Medical College Hospital, Peking Union Medical College and Chinese Academy of Medical Sciences, National Clinical Research Center for Dermatologic and Immunologic Diseases, Ministry of Science and Technology, State Key Laboratory of Complex Severe and Rare Diseases, Key Laboratory of Rheumatology and Clinical Immunology, Ministry of Education, Beijing, China; ^2^ Department of Nephrology, Peking Union Medical College Hospital, Peking Union Medical College and Chinese Academy of Medical Sciences, Beijing, China

**Keywords:** refractory lupus nephritis, leflunomide, induction therapy, drug safety, systemic lupus erythematosus

## Abstract

**Introduction:**

The condition of refractory lupus nephritis (LN) negatively affects the prognosis and life expectancy of the patients, posing a challenge to manage in clinical. This interventional study evaluated the efficacy as well as safety of leflunomide in patients with refractory LN.

**Methods:**

Twenty patients with refractory LN were enrolled in this study. A daily dose of 20–40 mg of leflunomide was given to the patients orally. Meanwhile, immunosuppressives were withdrawn, and corticosteroids were gradually tapered. There was an average follow-up period of 3, 6, and 12 months for most patients while some were observed for as long as 24 months. We recorded biochemical parameters and side effects. We calculated the response rate using intention-to-treat analysis.

**Results:**

Eighteen patients (90%) completed the study. At 3 months, 80% (16/20) of the patients achieved more than a 25% decrease in 24-hour urine protein quantity. At 6 months, three patients (15%) achieved a partial response, and five patients (25%) achieved a complete response. However, by 12 months and 24 months, the complete response rate dropped to 15% and 20%, respectively. The objective responses were 30% (6/20), 40% (8/20), 40% (8/20), and 30% (6/20) at 3, 6, 12, and 24 months, respectively. Two patients withdrew from the study due to developing cytopenia and leucopenia.

**Conclusion:**

In patients diagnosed with refractory LN, our study shows that leflunomide could be a promising treatment option owing to its response rate and safety profile.

## 1 Introduction

Lupus nephritis (LN) is one of the most common manifestation of systemic lupus erythematosus (SLE), characterized by autoantibody-induced immune complex formation and systemic inflammation, leading to kidney damage. Nearly half of the patients diagnosed with SLE also suffer from LN, one of the causes of their mortality (1). Despite aggressive immunosuppressive treatment, a great proportion of patients with LN respond poorly, especially those with refractory LN. Hence, the therapy choice for patients diagnosed with refractory LN presents a great challenge in a clinical setting.

Leflunomide (LEF), a dihydroorotate dehydrogenase inhibitor, targets lymphocytes with antiproliferative activity, preventing inflammation by suppressing the expression of pro-inflammatory molecules and activating cell-cell contact (2). In a retrospective observational study, LEF treatment for SLE appeared to be safe and effective, particularly for joint symptoms, and resulted in improved Physicians’ Global Assessment and SLE Disease Activity Index (2). The effects of LFN on LN have been demonstrated in a wide range of experimental models and clinical studies. According to a meta-analysis which included 11 randomized controlled trials (RCT), the efficacy of LEF was equal to that of cyclophosphamide (CTX) (total remission relative risk = 1.20). However, LEF had a better safety profile, showing fewer adverse drug reactions (3). Despite this, data supporting the use of LEF for refractory LN are limited. A network meta-analysis revealed that LEF, combined with CTX and glucocorticoids, resulted in significantly higher overall responses than traditional CTX and glucocorticoid treatment regimens (odds ratio = 3.05; 95% CI = 1.05–8.84). According to the study by Tam et al, patients with LN who were refractory to conventional immunosuppression or intolerant to it were treated with LEF, 47% (8/17) achieved a partial response (PR) and 29% (5/17) achieved a complete response (CR) (4).

Recently, according to a randomized trial with long follow-up period, in terms of maintenance therapy, LEF was proven to be non-inferior to azathioprine in patients with LN (5). Based on this evidence, there is reason to believe that LEF might be a promising choice in treating refractory LN. Therefore, the purpose of this study was to determine whether LEF could be effective and safe in the management of refractory LN.

## 2 Materials and methods

### 2.1 Participants

Between July 2015 and January 2019, 20 patients diagnosed with refractory LN were recruited in this exploratory intervention study at the Peking Union Medical College Hospital. The inclusion criteria included: diagnosis of SLE (defined by the 2012 Systemic Lupus International Collaborating Clinics criteria) (6) and refractory LN (defined as proteinuria and/or estimated glomerular filtration rate (eGFR) not improved or worsened for 4–6 months two different induction regimens according to standards of care, based on the KDIGO and EULAR/ERA-EDTA guidelines) (7–9). Patients who did not adhere to the prescribed therapy, and those with any history of malignant diseases, severe allergy reaction, severe hepatic insufficiency, pregnancy status, lactation status, or childbearing intention, were excluded. Repeated renal biopsy was recommended to each suitable patients without contraindication. The sample size is calculated with reference to the preliminary study (4) and based on the sample size calculation formula. The usage and dosage of the renin angiotensin system inhibitors (RASi) has been consistent pre and post the study. Ethical approval was obtained from the Research Ethics Committee of the Peking Union Medical College Hospital (No. K2683). Informed consent is taken from all the participants present in the study.

### 2.2 Treatment regimen

A daily dose of 20–40 mg of LEF (Cinkate Corporation, Beijing, China) was administered to the patients orally for 1–2 years. Meanwhile, previous treatments with corticosteroids and hydroxychloroquine were gradually tapered according to standardize CS tapering protocol (8, 9) to the minimal dose required to maintain remission (2.5-5mg/d prednisolone-equivalent). The previous immunosuppressive medications have all been discontinued. There was an average follow-up period of 3, 6, and 12 months for most patients while some were observed for as long as 24 months. Clinical manifestations, medication, and laboratory data (complete hemogram, 24-hour urine protein quantity, renal function test, lymphocyte subsets, and complements) were collected during follow-up.

### 2.3 Biochemical measurements

Clinical and laboratory data were obtained from the patients’ medical records. Levels of anti-double stranded DNA antibodies and antinuclear antibody were measured using immunofluorescence assays. Additionally, 24-hour urine protein (24h UP) quantity, urine test, albumin, urea, serum creatinine (sCr), C3 and C4 levels, serum immunoglobulin, inflammation parameters (including levels of high-sensitivity C-reactive protein and erythrocyte sedimentation rate), and a complete hemogram were measured. Serum samples were collected after an 8-hour fasting period in the morning.

### 2.4 Response criteria

The primary end point was the response of patient. It was evaluated 3, 6 months, and 1 year after enrollment, with 13 patients continuing the treatment for 2 years. For LN, the response criteria were: A) CR defined as improved or stabilized kidney function (a sCr change within ±25% of the baseline) and a 24h UP quantity <0.5–0.7 g per day; B) PR defined as a markedly improvement in 24-hour UP quantity (more than a 50% decrease to <3 g per day if the baseline 24hUP was >3.5 g per day or to ≤1 g per day if the baseline urine protein did not reach the nephrotic syndrome level), serum albumin ≥30 g/L, and stable or improved kidney function (sCr change was within ±25% of baseline value); C) No response (NR), defined as patients without CR or PR (8, 10). The percentage of more than a 25% decrease in 24-hour urine protein quantity was calculated as the secondary endpoint. Patients with CR or PR were considered to have an overall response (OR) to therapy. The term “treatment failure” refers to either an ineffective response or the need for further immunosuppressant therapy (4). We calculated the response rate using intention-to-treat analysis.

### 2.5 Statistical analyses

All statistical analyses were performed with R statistical software (version 3.4.3; http://www.R-project.org/) and SPSS software (version 20.0; SPSS Inc., Chicago, IL, USA). For normal distributions of continuous variables, data are presented as mean + standard deviation (SD). For skewed distributions of continuous variables, the average and interquartile range (IQR) are presented. For categorical variables, data are presented as numbers and proportions (%). Data between groups were compared using the Student’s *t*-test for continuous variables. Correlation analysis was performed using Pearson’s correlation coefficient, and statistical significance was set at p<0.05.

## 3 Results

### 3.1 Clinical and laboratory characteristics of the participants

This study recruited 20 patients diagnosed with refractory LN. Eight patients underwent renal biopsy, with six LN class IV, one LN class VI+V, and one LN class V [according to 2018 International Society of Nephrology/Renal Pathology Society classification (11)]. Renal biopsy was not performed in the rest of patients due to contraindications (thrombocytopenia, moderate to severe anemia, taking anticoagulations, etc.) or personal reasons after a detailed discussion of the pros and cons with the patients. Inclusion of patients had a mean age of 28.1 ± 8.9 years, and 80% of them were female. There was a range of 3-16 years of disease duration in SLE (average = 8.2 years, IQR = 5.0–10.0 years), and the mean disease duration in LN was 6.1 years (range = 1–16 years, IQR = 3.0–8.8 years). LN-related clinical manifestations begin with edema, hematuria, proteinuria, and/or abnormal renal function. Other manifestations included arthritis (80%), malar rash (55%), fever (35%), anemia (40%), and thrombocytopenia (35%). The baseline characteristics of the participants are presented in **Table 1**.

**Table 1:** Basic characteristics and adverse events of LEF for refractory LN.

No.	Sex	Age, years	Disease duration, years	Renal involvement duration, years	Auto-antibodies	Previous immuneuppressive agents	RASi	Proteinuria (g/24h)	Serum creatine (*µ* mol/L)	CS dosage at enrolment (prednisolone-equivalent, mg/d)	Adverse events
1	F	27	13	7	ANA, anti-Sm, anti-RNP, anti-dsDNA, ACL	CTX, MMF, AZA	losartan	7.16	53	10	
2	M	23	10	5	ANA, anti-dsDNA, anti-rRNP, anti-SSA, anti-RNPm AnuA, AHA, p-ANCA	CTX, CsA, FK506, MMF	Irbesartan	7.40	89	10	
3	F	32	16	16	ANA, anti-RNP, anti-SSA, anti-dsDNA, anti- Sm, anti-Ro52, AHA, AnuA, ACL, anti-β2GPI	CTX, FK506, MMF	Irbesartan	4.83	70	10	
4	M	25	10	7	ANA, anti-RNP, anti-Sm	CTX, CsA, FK506, MMF, AZA	Irbesartan	4.36	96	10	
5	F	28	8	8	ANA, anti-dsDNA	CTX, FK506, MMF	Irbesartan	2.19	99	15	Cytopenia
6	F	25	4	1	ANA, anti-dsDNA, anti-SSA, AHA	CTX, CsA, MTX, MMF	Fosinopril	1.4	84	10	
7	M	26	9	9	ANA, anti-dsDNA, anti-Sm, anti-RNP, ANuA, AHA, LA	CTX, FK506, MMF, AZA	Fosinopril	12.56	63	5	
8	F	40	13	9	ANA, ant-dsDNA, anti-SSA, anti- SSB, AHA, ANuA	CTX, CsA, FK506, MMF, AZA	losartan	4.46	87	50	
9	F	30	5	5	ANA, anti-dsDNA, anti- Sm, anti-rRNP, ANuA, AHA, anti-SSA	CTX, MMF	losartan	4.51	94	5	
10	F	23	7	3	ANA,	CTX, MMF	Fosinopril	4.47	47	15	
11	F	17	6	1	ANA, anti-dsDNA, anti- Sm, anti-rRNP, ANuA, anti-RNP, anti-SSA	CTX, FK506, MMF	Benazepril	1.51	39	30	
12	F	16	5	5	Anti-dsDNA, anti-rRNP, LA	FK506, MMF	/	2.99	62	5	
13	M	15	3	3	ANA, anti-dsDNA, anti-Sm, anti-RNP, AHA, LA, ACL, anti-β2GPI	CTX, MTX	Irbesartan	1.91	84	2.5	
14	F	33	5	1	ANA, anti-dsDNA, anti- Sm, anti-RNP, ANuA, AHA, anti-SSA, anti-SSB, anti- Ro52	MTX, FK506, MMF	Fosinopril	1.32	61	10	
15	F	30	13	13	ANA,, anti-dsDNA	CTX, FK506, MMF	Irbesartan	2.31	67	15	
16	F	34	5	4	ANA, anti-dsDNA,, anti-RNP, ANuA, AHA, anti-SSA, anti-β2GPI	CTX, MMF	losartan	6.07	69	30	cytopenia
17	F	23	10	10	ANA, anti-dsDNA, anti- SSA, anti-rRNP, anti-Ro52	CTX, MMF, AZA	Irbesartan	4.10	56	30	
18	F	53	6	6	ANA, anti-dsDNA, ANuA	CTX, MMF, AZA	Irbesartan	3.05	70	5	
19	F	38	7	7	ANA, anti-dsDNA, anti-Sm, anti-RNP, anti-Ro52	CTX, MMF	Fosinopril	2.16	72	15	
20	F	23	9	1	ANA, anti-dsDNA, LA, ACL, anti-β2GPI	CTX, CsA, MTX, MMF	/	2.39	74	40	

The previous treatments were for all the manifestations of SLE, including LN.

ANA, Anti-nuclear antigen; ACL, anti-cardiolipin; IVIG, intravenous immunoglobulin; CS, corticosteroids; CsA, cyclosporine A; CTX, cyclophosphamide; MMF, mycophenolate mofetil; AZA, azathioprine; MTX, methotrexate; HCQ, hydroxychloroquine; RASi, renin angiotensin system inhibitors; F, female; M, male; /, none.

### 3.2 Therapy response rate

**Figure 1:**
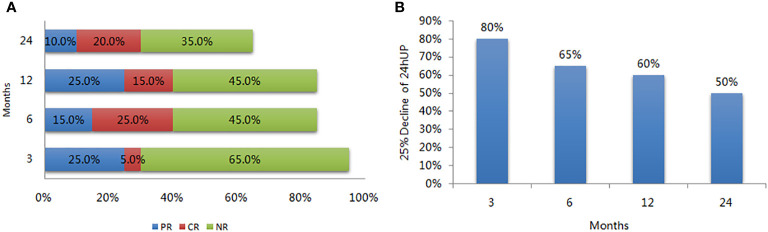
Response rate of LEF in patients with refractory lupus nephritis. **(A)** Rate of partial response (PR), complete response (CR), and no response (NR) during follow-up; **(B)** The ratio of more than 25% decrease in the 24-hour urine protein during follow-up.

Eighteen patients (90%) completed the study, and 13 completed the 2-year follow-up. At 3 months, 25% patients (5 out of 20) achieved PR; however, the PR rate fluctuated, measuring at 15%, 25% and 10% at the 6-, 12- and 24-month periods, respectively. The ratio of patients with more than a 25% decrease in their 24-hour urine protein quantity was 80% after 3 months. Five patients (25%) achieved CR at 6 months; however, the CR rate dropped to 15% after 12 months and achieved 20% after 24 months (One of the patient who achieved CR at month 6 and month 24 missed the month 12 follow-up due to COVID-19 pandemic outbreak). Treatment failure was observed in 9 patients (9/18) after 12 months, as well as in 7 of the remaining 13 patients after 24 months. The OR rates were 30%, 40%, 40%, and 30% at 3, 6, 12, and 24 months, respectively. As for the secondary endpoint, the ratio of patients with more than a 25% decrease in their 24-hour urine protein quantity was 80% at month 3, 65% at month 6, 60% at month 12, and 50% at month 24 in the intention-to-treat analysis (**Figure 1**).

### 3.3 Monitoring of laboratory parameters

**Figure 2:**
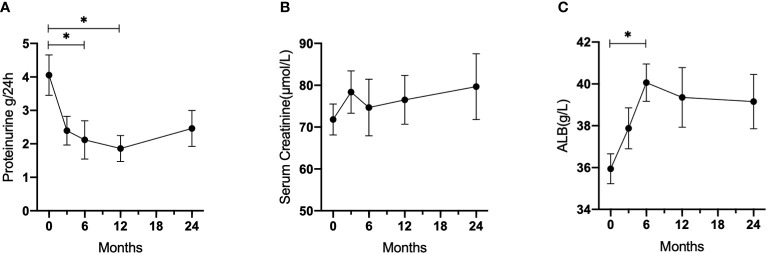
Laboratory parameters changes during follow-up in patients with refractory LN who received LEF. **(A)** 24-hour urine protein. **(B)** Serum creatinine. **(C)** Albumin. *p < 0.05.

**Figure 2** summarizes the changes in proteinuria, serum creatinine, and albumin levels in all patients. The 24-hour urine protein levels decreased significantly from 4.06 ± 2.71 to 2.11 ± 2.36 g per day after 6 months and 1.66 ± 1.46 g per day after 12 months (p = 0.013 and 0.006, respectively), while increasing slightly to 2.46 ± 2.09 g per day after 24 months (p = 0.140). The serum creatinine levels elevated over time (71.8 ± 16.6 μmol/L after 6 months, 74.7 ± 28.7 μmol/L after 12 months, and 79.7 ± 30.5 μmol/L after 24 months), but these changes were not statistically significant. Mean serum albumin increased from 35.9 ± 3.0 g/L at baseline to 40.1 ± 3.6 g/L after 6 months (p = 0.0367), 39.4 ± 5.3 g/L after 12 months (p = 0.1469), and 39.2 ± 4.7 g/L after 24 months (p = 0.2120). However, the changes in serum C3, C4, IgG, IgM, and IgA levels were not statistically significant when compared to baseline levels.

### 3.4 Factors related to response rate

Univariate analysis was performed to analyze factors affecting the response rate at 12 and 24 months (**Table 2**). Patients with lower albumin levels (<35 g/L) and lower eGFR (<90 mL/min/1.73 m^2^) at 12 months seemed to benefit more from LEF, although this relationship was not statistically significant. No other associations were statistically significant at 24 months.

**Table 2:** Factors related with OR at 12 months and 24 months.

	12M			24M		
	OR	non-OR	P value	OR	non-OR	P value
Male	1 (12.5%)	3 (33.3%)	0.576	0	3 (42.9%)	0.192
Renal DD ≥3 years	6 (75%)	5 (55.6%)	0.620	4 (66.7%)	4 (57.1%)	0.725
Fever	3 (37.5%)	4 (44.4%)	1.000	0	3 (42.9%)	0.192
Skin involvement	6 (75%)	9 (100%)	0.206	5 (83.3%)	7 (100%)	0.462
Neural system involvement	1 (12.5%)	2 (22.2%)	1.000	2 (33.3%)	2 (28.6%)	1.000
Joints involvement	7 (87.5%)	8 (88.9%)	1.000	5 (83.3%)	6 (85.7%)	1.000
Hematological involvement	5 (62.5%)	8 (88.9%)	0.294	4 (66.7%)	6 (85.7%)	0.559
Serosal effusion	3 (37.5%)	1 (11.1%)	0.294	2 (33.3%)	1 (14.3%)	0.559
24hUP ≥3.5 g/d	4 (50%)	5 (55.6%)	1.000	3 (50%)	4 (57.1%)	1.000
Abnormal sCr	0	2 (22.2%)	0.156	0	1 (14.3%)	1.000
ALB<35g/L	4 (57.1%)	1 (11.1%)	0.106	2 (40%)	1 (14.3%)	0.559
eGFR < 90ml/min/1.73m^2^	0	4 (44.4%)	0.082	0	3 (42.9%)	0.192
Low C3	5 (62.5%)	4 (44.4%)	0.637	4 (66.7%)	2 (28.6%)	0.170
Low C4	4 (50%)	2 (22.2%)	0.335	4 (66.7%)	1 (14.3%)	0.103
Low IgG	3 (37.5%)	3 (33.3%)	1.000	2 (33.3%)	2 (28.6%)	1.000
Low WBC	2 (25%)	2 (22.2%)	1.000	3 (50%)	0	0.070
Low lymphocyte counts	1 (12.5%)	1 (11.1%)	1.000	1 (16.7%)	0	0.462
Anemia	1 (12.5%)	2 (22.2%)	1.000	1 (16.7%)	1 (14.3%)	1.000

DD, disease duration; 24hUP, 24-hour urine protein; sCr, serum creatinine; ALB, albumin; eGFR, estimated glomerular filtration rate; C3, complement 3; C4, complement 4; IgG, immunoglobulin G; WBC, white blood cell.

### 3.5 Safety

The follow-up period included a review of all patients’ symptoms and laboratory indicators of side effects. Adverse events, such as cytopenia and leucopenia, were observed in two patients (Grade 3 and 2, Common Terminology Criteria for Adverse Events version 5.0). The average difference in lymphocyte counts was not statistically significant (1.64 ± 1.34×10^9^/L at baseline; 1.77 ± 1.26×10^9^/L at 3 months; 1.86 ± 1.20×10^9^/L at 6 months; 2.01 ± 1.31×10^9^/L at 12 months; 1.89 ± 1.04×10^9^/L at 24 months; p = 0.89). Abnormal blood cell counts were measured after LEF withdrawal. There were no deaths, severe infections, or malignancies observed in the participants in the study.

## 4 Discussion

The clinical management of patients with refractory LN remains a significant challenge. This interventional study recruited 20 participants receiving LEF in combination with corticosteroids as induction therapy for refractory LN, with a long observation period and strict selection criteria. The main findings showed that LEF could be an alternative treatment option for patients with refractory LN.

Kidney involvement is common in SLE, as nearly half of all patients with SLE develop LN during the disease duration (12). LN is a common cause leading to end-stage renal disease and mortality in patients with SLE (13). It has been reported that 14-33% of patients with LN had nos respond to aggressive immunosuppression even after prompt diagnosis and treatment (14, 15). Failure to achieve CR after 2 years, and the time required to achieve CR, are two important risk factors for poor renal outcomes (16, 17). Refractory LN presents great challenges in clinical practice; hence, it is imperative to immediately and rigorously control intrarenal inflammation with effective induction therapy in order to minimize nephron loss (18). The optimal treatment regimens for refractory LN have not been investigated. Moreover, studying refractory LN presents with many difficulties. First, a consensus definition of refractory LN has not yet been reached, which brings substantial heterogeneity between different studies. Second, there is also no consensus definition of renal response in patients with refractory LN. Moreover, patients present with heterogeneity in genetic background, LN manifestation, and kidney histopathology; however, there is a low ratio of patients with a repeat kidney biopsy. Strict inclusion criteria were developed in this study, s. The observation period should also be sufficient for the previous standard treatment plan to rule out patients with late responses. Two different standard-of-care induction regimens were used, and patients with a history of non-adherence to their prescribed treatment were excluded to avoid misclassification as refractory LN due to poor medication adherence.

LEF can inhibit *de novo* pyrimidine nucleotide biosynthesis, prevent DNA synthesis, arrest the proliferation of T lymphocytes, and decrease autoimmune responses (19). It can also inhibit tyrosine kinase activity by inhibiting the proto-oncogene c-Src pathway (20). Several studies have shown that LEF can be effective in treating SLE and LN. In a study involving MRL/lpr mice, treatment with LEF resulted in fewer autoantibodies and immune complex deposits in mouse glomeruli (21). In clinical trials, patients with LN receiving LEF in combination with prednisone displayed no inferior efficacy or safety to those receiving CTX as induction therapy and azathioprine as maintenance therapy (5, 22). In a meta-analysis comparing LEF and CTX, in spite of similar results reported on the SLE Disease Activity Index, LEF showed an improved safety profile and efficacy for the treatment of LN (3). Data supporting the use of LEF as an alternative therapy for refractory LN are scarce. Tam et al. (4) treated 17 patients with LN who had refractory disease or could not tolerate conventional immunosuppression with LEF. Moreover, 13/17 (76%) patients achieved an objective response (29% with CR and 47% with PR) after 48 weeks. However, despite the small sample size, the selection criteria were not met. Seven patients had contraindications to the standard therapy, and the “refractory” patients had a short observation period. Due to insufficient studies with long-term follow-up and high-level evidence, the KDIGO and EULAR/ERA-EDTA guidelines did not recommend LEF as a first-line induction therapy (8, 9). Therefore, the clinical application of LEF for LN treatment has not been widely studied, and further evaluation is required before recommending LEF as a treatment for refractory LN.

In the intention-to-treat analysis of the study, 40% of the patients responded to LEF at 12 months, and a response rate of 30% was reported at 24 months. The OR rates were not superior to those of other immunosuppressants or biologics reported in other studies of refractory LN. Rituximab (RTX) has shown efficacy in many uncontrolled and open-label observational studies in patients with refractory LN (with different diagnostic criteria in each study), reflected by OR rates ranging from 53–94.1% (23–30). Thus, from an expert perspective, RTX is recommended as the first-choice treatment for refractory LN (7). However, as an off-label treatment choice for LN, RTX is expensive for most patients, limiting its clinical use. Multitarget therapy (mycophenolate mofetil plus tacrolimus) resulted in good OR rates for refractory LN, ranging from 55.5–70% (31, 32). However, the sample size in these studies was small (only 12 and 6 patients, respectively), and this may influence the credibility of the results. Only few patients have reported to receive some alternative therapies for refractory L, including anti-plasma cell therapy (bortezomib, daratumumab) (33, 34), CD19-targeted chimeric antigen receptor T cells (35), low-dose IL-2 therapy (36), and hematopoietic stem cell transplantation (37). Moreover, the definitions of refractory LN and response were inconsistent across these studies; hence, the results should be interpreted with caution. The strict selection criteria for recruiting challenging patients in clinical practice was a strength of our study, leading to a relatively low OR rate. Thus, comparing the OR rates between different studies is not justified.

LEF can be an alternative treatment when the use of multiple routine immunosuppressants has become ineffective. In this study, the promising effect of LEF was observed on reducing the 24-hour urine protein quantity by more than 25%, especially after 3 months. Our results indicated that LEF might be helpful in the induction period and could be a treatment choice for multitarget therapy. Furthermore, patients with severe refractory LN, for example, those with lower albumin levels (less than 35 g/L) and lower eGFR (less than 90 mL/min/1.73 m^2^) at 12 months, appeared to benefit more from the therapeutic effects of LEF. However, we were unable to detect all predictive factors and mechanisms due to the small sample size in this study.

It is important to evaluate the safety of newly developed therapeutic regimens. LEF is basically well-tolerated, with mild adverse effects easily to be managed, such as liver damage, hypertension, diarrhea, and peripheral neuropathy. The incidence of side effects was reported to be 22.5–56.5% in patients treated with LEF (5, 38). In this study, two patients withdrew because of cytopenia and leucopenia even though the side effects were relatively mild, and both patients recovered soon after drug withdrawal. This study found a similar incidence of adverse effects as previous studies, but the safety of LEF for refractory LN needs further evaluation in a larger sample size study.

There remain several difficulties in the effective treatment of refractory LN. Clinicians must consider effectiveness, safety, cost, insurance policy mechanics, convenience, accessibility, and individual patient differences before making clinical decisions. Ideally, a repeat renal biopsy is required before decision-making in patients refractory LN (39). Compared to the immunosuppressants mentioned in previous studies, LEF is a promising, non-inferior candidate for induction treatment against LN with long-term safety. LEF treatment also has other advantages, such as easy accessibility, avoidance of hospitalization or outpatient infusion, and cost-effectiveness, especially when compared with multitarget therapies and biologics. However, some treatment attempts using LEF are still off-label. As an immunosuppressant with different mechanisms of action in LN, LEF is also a candidate for second-line or multitarget therapy. Since treatment needs for LN remain unmet in the clinic, there will always be some individual circumstances that would favor the choice of an alternative drug, such as LEF. Overall, our findings have both practical and clinical implications, providing evidence for the potential use of LEF in the therapy of refractory LN.

Several factors might have limited our analyses. First, being an exploratory interventional study and using strict inclusion criteria, only 20 patients were observed. Second, some data were lost owing to the COVID-19 pandemic, which resulted in several challenges for follow-up and laboratory tests. Hence, further RCTs are required to verify our findings. Moreover, two patients withdrew from the study because of cytopenia, and results were potentially minimized after using an intent-to-treat analysis. Finally, the eGFRs of all participants were above 60 mL/min/1.73 m^2^. Although data for refractory LN patients with insufficient kidney function are limited, confounders from the differential diagnosis of late irreversible kidney damage and treatment failure were also avoided.

In conclusion, refractory LN requires further research to develop effective treatment strategies. This study showed that, for patients with refractory LN, LEF could be a promising treatment option because of its response rate and acceptable safety.

## Data availability statement

The original contributions presented in the study are included in the article/supplementary material. Further inquiries can be directed to the corresponding authors.

## Ethics statement

The studies involving human participants were reviewed and approved by Research Ethics Committee of the Peking Union Medical College Hospital. Written informed consent to participate in this study was provided by the participants’ legal guardian/next of kin.

## Author contributions

Conceptualization: SZ, LW, ML. Data curation: SZ, LW. Formal analysis: SZ, YC, XC. Funding acquisition: SZ, LW. Investigation and methodology: SZ, LW, YZ, XZ, FZ. Supervision: XZ, FZ. Visualization: SZ, XC. Roles/writing - original draft: SZ. Writing - review and editing: YC, XC, YZ, XZ, FZ, LW, ML. All authors contributed to the article and approved the submitted version.
